# The Burden of Invasive Bacterial Disease and the Impact of 10-Valent Pneumococcal Conjugate Vaccine in Children <5 years hospitalized for Meningitis in Lusaka, Zambia, 2010–2019

**DOI:** 10.1093/infdis/jiab193

**Published:** 2021-09-01

**Authors:** Kaunda Yamba, Evans Mpabalwani, Ruth Nakazwe, Evans Mulendele, Goitom Weldegebriel, Jason M Mwenda, Reggis Katsande, Linda de Gouveia, Elizabeth Chizema-Kawesha, Raphael Chanda, Belem Matapo, James C L Mwansa, Chileshe Lukwesa-Musyani

**Affiliations:** 1 University Teaching Hospitals, Pathology & Microbiology Laboratory, Lusaka, Zambia; 2 University of Zambia, School of Medicine, Department of Paediatrics & Child Health, Lusaka, Zambia; 3 University Teaching Hospitals, Children’s Hospital, Lusaka, Zambia; 4 World Health Organisation, Regional Office for Africa, Brazzaville, Republic of Congo; 5 Centre for Respiratory Diseases and Meningitis, National Institute for Communicable Diseases, Johannesburg, South Africa; 6 Ministry of Health, Directorate of Public Health, Disease Surveillance and Research, Zambia; 7 Lusaka Apex Medical University, Lusaka, Zambia

**Keywords:** *Streptococcus pneumoniae*, *Haemophilus influenzae*, *Neisseria meningitidis*, Pneumococcal conjugate vaccine, Zambia

## Abstract

**Background:**

Despite the availability of vaccines, invasive bacterial diseases remain a public health concern and cause childhood morbidity and mortality. We investigated the characteristics of etiological agents causing bacterial meningitis in children <5 years in the years pre- (2010–2012) and post- (2014–2019) 10-valent pneumococcal conjugate vaccine (PCV10) introduction in Zambia.

**Methods:**

*Streptococcus pneumoniae* (Spn), *Haemophilus influenzae* (Hi), and *Neisseria meningitidis* (Nm) from cerebrospinal fluid (CSF) were identified by microbiological culture and/or real-time polymerase chain reaction.

**Results:**

During the surveillance period, a total of 3811 children were admitted with suspected meningitis, 16% (598 of 3811) of which were probable cases. Bacterial meningitis was confirmed in 37% (221 of 598) of the probable cases. Spn *pneumoniae*, Hi, and Nm accounted for 67% (148 of 221), 14% (31 of 221), and 19% (42 of 221) of confirmed cases, respectively. Thirty-six percent of pneumococcal meningitis was caused by 10-valent pneumococcal conjugate vaccine (PCV10) serotypes, 16% 13-valent pneumococcal conjugate vaccine and 39% by nonvaccine serotype (NVS). There was an association between the introduction of PCV10 vaccination and a decrease in both Spn meningitis and the proportion of PVC10 serotypes in the postvaccination period. Antimicrobial susceptibility of 47 Spn isolates revealed 34% (16 of 47) penicillin resistance. The 31 serotyped Hi accounted for 74% type b (Hib) and 10% type a (Hia). All 42 serogrouped Nm belonged to serogroup W.

**Conclusions:**

There was a decline in pneumococcal meningitis and proportion of PCV10 serotypes in the postvaccination period. However, the serotype replacement with non-PCV10 serotypes and penicillin resistance warrant continued surveillance to inform policy.

Infectious diseases remain a leading cause of death globally in children below the age of 5 years [1]. In 2019, 5.2 million deaths were recorded in children ≤5 years with sub-Saharan Africa recording the highest at 2.8 million (53%) [1].

Although childhood vaccination prevents 2 million deaths a year worldwide, deaths caused by vaccine-preventable diseases among children aged ≤5 years is approximately 2.5 million deaths a year in Africa and Asia [2, 3]. *Streptococcus pneumoniae* ([Spn] pneumococcus), *Haemophilus influenza* (Hi) type b (Hib), and *Neisseria meningitidis* ([Nm] meningococcus) are the 3 main pathogens known to cause morbidity and mortality from acute bacterial meningitis in low-income countries [4, 5]. In 2011, before the introduction of pneumococcal conjugate vaccine in Zambia, a review of laboratory data to determine the main causative agents of meningitis at the University Teaching Hospital (UTH) found *S pneumoniae* to be the leading cause of bacterial meningitis [6].

Pneumococcal disease remains a public health concern because it is associated with long-term sequelae and high case-fatality rates (CFRs), which worsen by the rise in resistance to commonly used and affordable antibiotics such as penicillin [7, 8]. An estimated 1.6 million children under 5 years of age die every year as a result of pneumococcal disease, the highest burden being in sub-Saharan African countries with high human immunodeficiency virus (HIV) prevalence [8, 9]. More than 95 pneumococcal serotypes have been identified, based on differences in the antigenic characteristics of their capsular polysaccharides. These play a vital role in the pathogenesis, virulence, target organs, antimicrobial susceptibility, age, and geographical distribution of invasive pneumococcal disease [10]. Currently, 2 pneumococcal vaccines are available for children, namely, 10-valent pneumococcal conjugate vaccine (PCV10) and 13-valent pneumococcal conjugate vaccine. The PCV10 covers serotypes 1, 4, 5, 6B, 7F, 9V, 14, 18C, 19F, 23F, whereas PCV13 additionally covers serotypes 3, 6A, 19A.

In 2015, a global estimate of 29 500 Hib deaths occurred in HIV-uninfected children with an additional 1000 deaths in HIV-infected children [11]. The CFR ranged from 5% (with proper treatment) to 60% (improper/incomplete treatment) with 20%–40% sequelae such as blindness and hearing loss in survivors [12, 13]. Before the introduction of Hib conjugate vaccine, Hib was the leading cause of nonepidemic bacterial meningitis worldwide with more than 90% of Hib disease occurring in children ≤5 years of age [13]. *Haemophilus influenzae* type b has almost been eliminated globally due to the highly effective protein-polysaccharide conjugate Hib vaccine, which has been available for over 20 years [14].

*Neisseria meningitidis* is associated with high fatality and frequency of neurological sequelae and has the potential to cause large epidemics. Although it is observed worldwide, the highest burden of disease is in the meningitis belt of sub-Saharan Africa [15]. Approximately 50 000 to 1 200 000 meningococcal cases are reported yearly, mostly during the dry season [15]. There are 12 meningococcal serogroups, but only 6 (A, B, C, W, X, and Y) are known to cause epidemics, with serogroup A being the most prevalent in Africa [15]. Vaccines against meningococcal disease are serogroup specific and confer varying degrees of protection and coverage duration [16]. Although major improvements in strain coverage has been made, there is still no universal meningococcal vaccine [17].

 The World Health Organization (WHO) recommended the inclusion of Hib and the PCV10 using a 3 primary-dose series at age 6, 10, and 14 weeks in childhood immunization programs [18]. The introduction of vaccines in countries within WHO African region, as part of their national childhood Expanded Programme on Immunisation, has resulted in a significant decline in invasive bacterial diseases (IBDs) caused by pneumococcus and Hib [6, 8, 19].

Zambia introduced Hib vaccine in January 2004 and PCV10 (3 + 0 schedule) [8] in July 2013. There is currently no meningococcal vaccine available in Zambia.

 Zambia has been part of the WHO coordinated Invasive Bacterial Diseases Paediatric Bacterial Meningitis (PBM) surveillance network since 2003. In this study, we aim to review PBM surveillance data in Zambia over a 10-year period, 2010–2019. The objective of this investigation was to describe confirmed cases of bacterial meningitis and their clinical presentation, pathogen, and serotype distribution in the pre- and post-PCV10 introduction and pneumococcal antimicrobial susceptibility patterns.

## METHODS

### Study Design and Case Definition

The study was part of the African PBM sentinel surveillance network initiated by the WHO and other immunization collaborates in 2001. It comprises a robust, hospital-based sentinel surveillance system that collects case-based information on clinically suspected and laboratory-confirmed bacterial meningitis cases among children ≤5 years of age who present to healthcare facilities. The case definitions of suspected, probable, and confirmed bacterial meningitis were used as per WHO guidelines [20].

The time periods were categorized as follows: prevaccine introduction 2010 to 2012, postvaccine period 2014 to 2019, and 2013 as the year of vaccine introduction. Data from the year of vaccine introduction (2013) was not included when comparing the prevaccine against the postvaccine period.

### Study Site

The UTH, a national referral hospital in the capital city of Lusaka, is the only Zambian sentinel site. The UTH is a highly specialized referral hospital with several specialty departments. The Children’s Hospital is part of the UTH and has a 400-bed capacity with over 10 000 admissions per year. Approximately 85% of cases received at the Children’s Hospital are referred from district hospitals for specialized care.

### Target Population

Sentinel site surveillance was conducted at the UTHs Children’s Hospital. The cases were identified according to the 2003 WHO-recommended standards for surveillance of selected vaccine-preventable diseases and standard operating procedures for bacterial meningitis surveillance [21].

### Cerebral Spinal Fluid Collection and Questionnaire

Children who met the case definition had a lumbar puncture performed aseptically by a doctor under the supervision of an experienced pediatrician. Clinical and demographic data were collected from children whose parent/guardian had given informed consent using a WHO standard case investigation form. Once cerebral spinal fluid (CSF) was collected, it was transported to the onsite Microbiology and Chemistry laboratories within 1 hour of collection.

### Conventional Analysis of Cerebral Spinal Fluid Specimens

 Two CSF sample tubes per patient were submitted to the clinical laboratory; 1 for cell count, Gram stain, and bacterial culture and a second for glucose and protein concentrations. Bacterial culture was performed using blood and chocolate agar (containing 5% sheep blood) and MacConkey agar (Oxoid, Basingstoke, UK). All plates were incubated for 18–48 hours at 35–37°C; blood and chocolate agar in 5% CO_2_ and MacConkey aerobically. Any residual CSF sample (of at least 50 µL) was aliquotted and stored in −70°C.

 Pathogen identification was done using conventional phenotypic methods such as colony morphology, growth requirements, Gram stain, and biochemical tests [22]. When available, the Pastorex meningitis kit was used for the rapid detection of Hib, pneumococcus, and meningococcus groups A, B, C, W, and Y antigens from CSFs supernatant of with a turbid appearance or leukocytosis >100 cells/mm^3^.

### Real-Time Polymerase Chain Reaction Detection of Pathogens

 At scheduled intervals, the stored CSF specimens and any corresponding isolates were sent to the Regional Reference Laboratory at the National Institute for Communicable Diseases in South Africa where all samples were tested and genotyped by a multiplex real-time polymerase chain reaction (PCR) for the detection of *H influenzae*, *N meningitidis*, and *S pneumoniae*. Identification of pneumococcal isolates received was done using Optochin susceptibility and bile solubility and serotyping of by Quellung reaction using specific antisera (Statens Serum Institute, Copenhagen, Denmark).

The MagNA pure 96 instrument (Roche) was used to extract the total nucleic acid from 200 µL of all CSFs received, whereas the Applied Biosystems 7500 Fast real-time PCR instrument (Applied Biosystems, Foster City, CA) was run for the molecular detection of meningitis targeting *ctrA*, *lytA*, and *hpd* genes for *N meningitidis*, *S pneumoniae*, *H influenzae*, respectively [23]. The amplification cycle involved an initial denaturation step at 95°C for 10 minutes, followed by 45 cycles of 95°C for 15 seconds and 60°C for 1 minute. Positivity for each of the targets was inferred using cycle threshold (Ct) values. Different Ct cutoff values were used to define positive, negative, and inconclusive results. The Ct cutoff for pathogen gene detection (Hi = *hpd*; Nm = *ctrA*; Spn = *lytA*) was ≤35. Polymerase chain reaction detection of *RNaseP* gene was done to determine sample integrity and the absence of any possible inhibitors, thus confirming true negative PCR results. Detection of the *RNaseP* gene was used to confirm true negative PCR results; samples with a Ct value ≥36 were reported as PCR inconclusive.

### Specific Capsule Serogrouping and Serotyping Polymerase Chain Reaction Assays

Real-time PCR genotyping/grouping was then performed on all samples that tested PCR positive for 1 of the 3 genes. Pneumococcal serotyping of *lytA*-positive samples consisted of 8 multiplex reactions and detected 38 serotypes (including all serotypes in PCV13). Samples negative for all 38 serotypes were reported as NEG38 or nonvaccine type [24, 25]. In instances in which the PCR target could not distinguish between vaccine and nonvaccine serotype (NVS), we reported such serotype (9V/9A, 18A/18B/18C/18F) as nonvaccine serotype. For meningococcal serogrouping, 2 multiplex reactions were run, and the target genes included *sacB*, *synD*, *synE*, *synG*, *xcbB*, and *synF* genes for serogroups A, B, C, W, X, and Y, respectively [26], whereas 3 multiplex reactions were run for *H influenzae* serotyping, targeting *acsB* (type a), *bcsB* (type b), *ccsD* (type C), *dscE* (type d), *ecsH* (type e), and *bexD* (type f) [27].

### Antimicrobial Susceptibility Testing 

Antimicrobial disc susceptibility testing was performed on viable, culture-positive (59%, 47 of 80) Spn isolates. The interpretation of results were done according to the Clinical and Laboratory Standards Institute guidelines [28]. Minimum inhibitory concentrations (MICs) were determined using E-tests (Oxoid, Basingstoke, UK); penicillin and ceftriaxone susceptibility was classified as ≤0.06 µg/mL and ≤0.5 µg/mL, respectively. *Streptococcus pneumoniae* ATCC strain 49619 was used for quality control purposes.

### Statistical Analysis

Data were entered into a custom database using Epi Info version 3.5.4 (CDC, Atlanta, Georgia) and was analyzed using SPSS statistical software version 20 (IBM, Chicago, illinois). Categorical variables are reported as proportion and percentage.

Using a significance of 0.05 (5%), 3 different statistical tests were used to determine significance. We used (1) the Pearson correlation to measure the strength and significance of the association between the clinical characteristics and the 3 pathogens isolated (Spn, Hi, Nm), (2) unpaired *t* test to measure the significant difference between the proportion of probable cases of males and females, and (3) analysis of variance (ANOVA) to measure the significant difference between the proportion of confirmed cases among the 3 age groups. The surveillance protocol was cleared by WHO and the Zambia Ministry of Health Ethical Review Committee.

## RESULTS

### Suspected, Probable, and Confirmed Bacterial Meningitis Cases

Between 2010 and 2019, 3811 suspected bacterial meningitis cases were reported: 16% (598 of 3811) of them were defined as probable cases, and 37% (221 of 598) were confirmed bacterial meningitis cases. The years 2010 to 2012 were the prevaccination period with a total of 1050 suspected cases, 17% (181 of 1050) probable cases, and 28% (51 of 181) confirmed bacterial meningitis cases, whereas the years 2014 to 2019 were the postvaccination period with 2178 suspected cases, 15% (326 of 2178) probable cases, and 35% (113 of 326) confirmed bacterial meningitis cases. The year of vaccine introduction (2013) had 583 suspected cases, 91 probable cases, and 57 confirmed cases (Table 1).

**Table 1.** National Vaccination Coverage Rate and Year of Admission of Children <5 Years With Suspected Probable and Confirmed Bacterial Meningitis by Culture and/or PCR at the UTH Children’s Hospital, 2010–2019

Admission Year	National Vaccination Coverage Rate		Suspected Cases	Probable Cases	Confirmed Bacterial Meningitis Cases by Culture and PCR			
					Confirmed Cases	*Streptococcus pneumoniae*	*Haemophilus influenzae*	*Neisseria meningitidis*
	Pentavalent	PCV10						
2010	97	-	268	43 (16%)	4 (9%)	4 (100%)	0 (0)	0 (0)
2011	95	-	292	58 (20%)	13 (22%)	13 (100%)	0 (0)	0 (0)
2012	91	-	490	80 (16%)	34 (43%)	29 (85%)	3 (9%)	2 (6%)
2013	93	29a	583	91 (16%)	57 (63%)	42 (74%)	5 (9%)	10 (18%)
2014	92	88	521	78 (15%)	41 (53%)	20 (49%)	9 (22%)	12 (29%)
2015	90	81	500	64 (13%)	29 (45%)	11 (38%)	7 (24%)	11 (38%)
2016	99	98	471	65 (14%)	19 (29%)	11 (58%)	5 (26%)	3 (16%)
2017	94	94	245	35 (14%)	12 (34%)	9 (75%)	1 (8%)	2 (17%)
2018	90	90	239	46 (19%)	8 (17%)	5 (63%)	1 (13%)	2 (25%)
2019	88	89	202	38 (19%)	4 (11%)	4 (100%)	0 (0)	0 (0)
Total	-	-	3811	598	221	148	31	42

Abbreviations: PCR, polymerase chain reaction; PCV10, 10-valent pneumococcal conjugate vaccine; UTH, University Teaching Hospital.

NOTES: (1) PCV10 introduction July 2013. National Vaccination Coverage Rate (percentage) reference: Ministry of Health of Zambia, April 2020, Expanded Programme on Immunisation. M. Silitongo, February 2019, Unpublished data. (2) Pentavalent: The 5-in-1 vaccine giving protection against diphtheria, tetanus, pertussis, polio, and Hib. (3) PCV10: The 10-valent pneumococcal conjugate vaccine giving protection against pneumococal diseases caused by 10 serotypes (1, 4, 5, 6B, 7F, 9V, 14, 18C, 19F, 23F).

aYear PCV10 was introduced.

### Characteristics and Clinical Signs of Study Participants

Although suspected and probable meningitis cases were mostly male at 54% (2074 of 3811) and 53% (317 of 598) compared with females at 46% (1737 of 3811) and 47% (281 of 598), respectively, there was no significant difference (*P* = .77). Most of the children were in the 0–12 months age group followed by 24–59 months and then 12–24 months (Table 2), with strong evidence showing a significant difference between the proportions of the confirmed cases among the 3 age groups (*P* = .00), and the post hoc test for difference between each group showing significance at 95% confidence interval on each of the groups.

**Table 2.** Clinical and Demographic Characteristics of Children <5 Years With Suspected PBM at the UTH Children’s Hospital, 2010–2019

Characteristics	Suspected Cases	Probable Cases	Confirmed Cases	*Haemophilus influenzae*	*Streptococcus pneumoniae*	*Neisseria meningitidis*
Age in Months						
0 < 12 months	2852	424 (15%)	140 (33%)	22 (16%)	101 (72%)	17 (12%)
12 to < 24 months	307	54 (18%)	19 (35%)	2 (11%)	14 (74%)	3 (16%)
24–59 months	652	120 (18%)	62 (52%)	7 (11%)	33 (53%)	22 (36%)
Total	3811	598 (16%)	221 (37%)	31 (14%)	148 (67%)	42 (19%)
Gender						
F	1737	281 (16%)	113 (40%)	17(15%)	76 (67%)	20 (18%)
M	2074	317 (15%)	108 (34%)	14 (13%)	72 (67%)	22 (20%)
Total	3811	598 (16%)	221 (37%)	31 (14%)	148 (67%)	42 (19%)
Presence of seizure	1116	130 (12%)	62 (48%)	13 (21%)	41 (66%)	8 (13%)
Altered consciousness	457	69 (15%)	41 (59%)	5 (12%)	30 (73%)	6 (15%)
Dehydration	386	69 (18%)	20 (29%)	1 (5%)	18 (90%)	1 (5%)
Bulging fontanel	388	60 (16%)	25 (42%)	6 (24%)	15 (60%)	4 (16%)
Neck stiffness	269	41 (15%)	25 (61%)	7 (28%)	8 (32%)	10 (40%)
Lethargy	38	1 (3%)	0 (0)	0 (0)	0 (0)	0 (0)
Meningitis rash	5	0 (0)	0 (0)	0 (0)	0 (0)	0 (0)
High respiratory rate	678	134 (20%)	51 (38%)	2 (4%)	37 (73%)	12 (24%)
Difficulty breathing	448	60 (13%)	28 (47%)	2 (7%)	21 (75%)	5 (18%)
Cough	421	73 (17%)	32 (44%)	4 (13%)	21 (66%)	7 (22%)
Chest in-drawing	53	6 (11%)	1 (17%)	0 (0)	0 (0)	1 (100%)
Stridor	17	2 (12%)	0 (0)	0 (0)	0 (0)	0 (0)

Abbreviations: PBM, pediatric bacterial meningitis; UTH, University Teaching Hospital.

NOTES: Meningitis rash: reddish pinprick lesions that progress to widespread petechial eruptions due to bleeding under the skin. High respiratory rate: 50 breaths/minute for infants and neonates, 40 breaths/minute for children aged 12–35 months, and 30 breaths/minute for children aged 36–60 months.

Children presented with a wide range of symptoms and signs. In the period under review, the most common clinical sign was the presence of seizures at 29% (1116 of 3811), and the least common sign was petechial rash at 0.1 (5 of 3811) (Table 2). Using Pearson’s correlation, Spn had a positive correlation with high respiratory rate with a significance of 0.5, whereas Hi and Nm both had a positive correlation with neck stiffness with a significance of 0.00.

### Etiological Agents

In 93% (3526 of 3811) of the CSF specimens processed, culture- and/or PCR-positive confirmed cases totaled 107 and 221, respectively (Spn—80 culture positive and 148 PCR positive; Hi—14 culture positive and 31 PCR positive; Nm—13 culture positive and 42 PCR positive). Polymerase chain reaction had a higher sensitivity for identification and/or detection of the 3 pathogens compared with culture (52% for the confirmed cases, 46% for Spn, 55% for Hi, and 69% for Nm).

**Figure 1.**
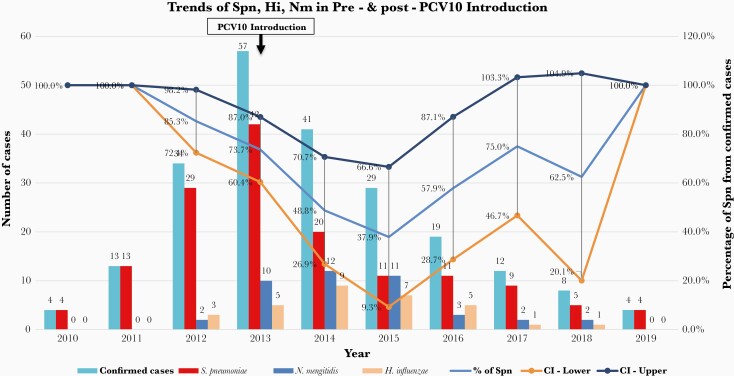
Trends of *Streptococcus pneumoniae* (Spn), *Neisseria meningitidis* (Nm), and *Haemophilus influenzae* (Hi) from 2010 to 2019, decline in Spn cases after PCV10 vaccine introduction in 2013, and the 95% confidence interval (CI) of the probability of Spn from confirmed cases.

 Of the 221 laboratory-confirmed meningitis cases, Spn (67%, 148 of 221) was the most common etiological agent followed by Nm (19%, 42 of 221) and Hi (14%, 31 of 221) (Table 1). *Streptococcus pneumoniae* showed a downward trend after PCV10 introduction in 2013 with 95% confidence interval of the probability of Spn from confirmed cases (the probability of having Spn from the confirmed cases in 2014 ranges from 26.9% to 70.7% and 28.7% to 87.1% in 2016) (Figure 1). Both Hi and Nm cases remained relatively low in the period under review; however, an increase in cases was seen in 2014 and 2015 (Figure 1).

### Serotypes

During the review period, we detected pneumococcal serotypes PCV10 (36%, 54 of 148), PCV13 (16%, 23 of 148), and NVS (39%, 57 of 148); however, a serotype was not possible from the remaining 9% (14 of 148) because the samples had insufficient deoxyribonucleic acid for serotyping. The predominant were serotypes were 1 and 6A/6B, which accounted for 14% and 11% of Spn cases. Serotype 6A/6B was included in the PCV13 group because PCV13 is known to cover both 6A and 6B; hence, the assumption of cross-protection was considered. The rare Spn serotype 46 detected during the 2012–2013 season was the most common among the NVS and accounted for 5% of all the Spn cases (Table 4).

**Table 4.** *Streptococcus pneumoniae* resistance profile to Penicillin and Ceftriaxone in Children <5 years at the UTH Children’s Hospital, 2010–2019

PCV10 Serotypes (n)	PEN MIC Range (µg/mL)	CRO MIC Range (µg/mL)
1 (10)	0.004 (S) to 0.06 (S)	0.008 (S) to ≤0.5 (S)
4 (1)	1 (R)	≤0.5 (S)
6B (2)	0.03 (S) to 0.06 (S)	≤0.5 (S)
6B (2)	0.25 (S) to 0.5 (R)	≤0.5 (S)
7F (1)	0.03 (S)	≤0.5 (S)
14 (1)	0.25 (R)	0.12 (S)
14 (1)	2 (R)	1 (I)
18C (2)	0.08 (S) to 0.015 (S)	0.015 (S) to 0.03 (S)
19F (5)	0.5 (R)	0.12 (S) to ≤0.5 (S)
23F (2)	0.03 (S) to 0.25 (R)	0.25 (S) to ≤0.5 (S)
Total (28)	**Resistance** **39% (11/28)**	**Intermediate 3.5% (1/28)**
Non-PCV10 serotypes (n)		
3	0.12 (R)	0.12 (S)
6A (1)	0.12 (R)	0.25 (S)
6A (1)	0.06 (S)	≤0.5 (S)
10F	0.06 (S)	≤0.5 (S)
15C (1)	0.06 (S)	≤0.5 (S)
15C (2)	0.12 (R)	≤0.5 (S)
18F (3)	0.008 (S)	0.015 (S)
19A (1)	0.25 (R)	≤0.5 (S)
46 (8)	0.008 (S) to 0.015 (S)	0.03 (S) to 0.015 (S)
Total (19)	**Resistance 26% (5/19)**	**Resistance (0)**

Abbreviations: CRO, cefotaxime; MIC, minimum inhibitory concentration; PCV10, 10-valent pneumococcal conjugate vaccine; PEN, penicillin; UTH, University Teaching Hospital.

NOTE: Non-PCV10 serotypes: serotypes not covered by PCV10.

 There was an association between the introduction of PCV10 vaccination and decrease in both Spn meningitis and the proportion of PVC10 serotypes in the postvaccination period. However, there was serotype replacement with NVS and unknown serotypes with a combination of vaccine serotype and NVS (Table 4).

During the 10-year period, serotyped Hi belonged to 74% (23 of 31) Hib and 10% (3 of 31) Hia, whereas the remaining 16% (5 of 31) were unable to be serotyped due to a high Ct. All of the *N meningitidis* (100%, 42 of 42) cases were serogroup W.

### Antimicrobial Susceptibility

Antimicrobial susceptibility testing was performed on the viable, culture-positive (59%, 47 of 80) Spn isolates; 46 of 47 were from the prevaccine period. Sixty-six percent (31 of 47) of pneumococcal isolates were susceptible to penicillin, 55% (17 of 31) of which were PCV10 serotypes and 45% (14 of 31) were non-PCV10 serotypes, with MICs ranging between 0.004 and 0.06 µg/mL. Susceptibility to ceftriaxone was 98% (46 of 47), 59% (27 of 46) of which were PCV10 serotypes and 41% (19 of 46) were non-PVC10 serotypes, with MICs ranging between 0.008 and ≤0.5 µg/mL. Of the 47 isolates tested, penicillin-resistant isolates 39% (11 of 28) were PCV10 serotypes and 26% (5 of 19) were non-PCV10 serotypes. Serotype 14 was intermediate to ceftriaxone and was the only isolate from the postvaccination period (Table 3). The high resistance to penicillin in PCV10 serotypes in comparison to non-PCV10 serotypes suggests that if the overall proportion of PCV10 serotypes decreases in the population as seen in our study (Table 4), resistance would presumably become less common.

**Table 3.** *Streptococcus pneumoniae* Serotypes Detected in Children <5 Years in the Pre- and Postvaccination Periods at the UTH Children’s Hospital, 2010–2019

Serotypes	Prevaccination Period (2010–2012)	Vaccination Year (2013)	Postvaccination Period (2014–2019)	Total
PCV10 Serotypes = 36% (54 of 148)				
*1*	9	7	5	21
*4*	2	2	0	4
*5*	0	0	1	1
*6B*	1	3	0	4
*7F*	1	0	0	1
*14*	0	2	2	4
*18C*	2	2	0	4
*19F*	2	4	1	7
*23F*	3	3	2	8
Total	20	23	11	54
PCV13 Serotypes = 16% (23 of 148)				
*3*	1	1	1	3
*6A*	1	1	0	2
*6A/6B*	1	2	13	16
*19A*	1	0	1	2
Total	4	4	15	23
Nonvaccine Serotypes = 39% (57 of 148)				
*8*	0	0	1	1
*9V/9A*	0	0	2	2
*10A/B*	1	0	0	1
*10F/10C/33C*	1	0	0	1
*15C*	3	0	0	3
*18F*	1	3	0	4
*18A/18B/18C/18F*	0	2	1	3
*19B/F*	3	0	0	3
*46*	7	1	0	8
NEG38	3	8	20	31
Total	19	14	24	57
Insufficient DNA—Unable to Obtain Serotype = 9% (14 of 148)				
Total	4	4	6	14
Overall total	47	45	56	148

Abbreviations: DNA, deoxyribonucleic acid; UTH, University Teaching Hospital.

NOTES: (1) PCV10: The 10-valent pneumococcal conjugate vaccine giving protection against pneumococal diseases caused by 10 serotypes (1, 4, 5, 6B, 7F, 9V, 14, 18C, 19F, 23F). (2) PCV13: Pneumococcal conjugate vaccine with purified capsular polysaccharide of 13 serotypes of Spn (1, 3, 4, 5, 6A, 6B, 7F, 9V, 14, 19A, 19F, 18C, and 23F). (3) Nonvaccine serotype: Serotypes not covered by PCV10 and/or PCV13.

## Discussion

To determine the burden of IBD in children ≤5 years, Zambia embarked on sentinel site meningitis surveillance, which provided data before and after PCV introduction. This surveillance has demonstrated that IBD is a public health problem in Zambia. For the first time, we have described the vaccine-preventable bacterial meningitis causative agents, clinical symptoms, and pneumococcal antimicrobial susceptibility patterns in Zambia. The decline in the frequency of probable bacterial and confirmed meningitis cases after PCV10 introduction is notable. The reduction of probable bacterial meningitis cases is consistent with findings by Mpabalwani et al [8], who observed substantial decline in meningitis and pneumonia hospitalizations among children aged <5 years in the first 3 years after PCV10 introduction in Zambia.

 In this study, we observed that *S pneumoniae* (67%, 148 of 221) was the most common causative pathogen of meningitis in children <5 years old. Our findings are comparable to findings in other developing countries such as Mozambique [29] and South Africa [30]. Likewise, these findings were consistent with a systematic review that looked at the Global Etiology of Bacterial Meningitis [31]. With the decline in the detection of *S pneumoniae* in the post-PCV introduction era, it is expected that meningitis and pneumonia cases, which are among the top 10 causes of hospital admission of children ≤5 years, would decline further [8].

 The predominant Spn serotypes that cause bacterial meningitis in children ≤5 were serotype 1 and 6A/6B (39%; 21 of 54). This finding was similar to what was found in Malawi [32] and in the United States [33] but different from the findings in Nigeria, where serotype 23F was the most prevalent [34]. The combination and distribution of serotype differs from country to country [9]. This finding can be attributed to the geographical differences in the distribution of pneumococcal serotypes [10]. Of note in this study is the high number of non-PCV10 vaccine serotypes confirming the occurrence of serotype replacement, as has been seen in many other countries after the introduction of a PCV vaccine [35–38]. This underscores the need for continuous surveillance to monitor changes in dominant circulating serotypes [39].

 Although this study did not include the prevaccine period for *H influenzae*, the detection of *H influenzae* remained relatively low at 14% (31 of 221), similar to the findings in Gambia where *H influenzae* (16%) was the lowest among confirmed meningitis cases [40]. Furthermore, our findings are consistent with another global systematic review that looked at the effects of Hib vaccine on childhood meningitis mortality and demonstrated that the majority of childhood meningitis mortality was prevented with the existing Hib vaccine [19].

 There was a slight increase in confirmed *N meningitidis* cases (19%, 42 of 221), and all cases belonged to serogroup W. This serogroup was not seen in Zambia until approximately 2010 and replaced serogroup A. After the introduction of MenAfriVac, several countries in sub-Saharan Africa recorded epidemics, and *N meningitidis* serogroup W was the leading cause [41]. The shift in the epidemiology of meningococcal serogroup distribution in Zambia is similar to other nearby countries such as South Africa, Madagascar, and Mozambique [42–44]; however, it was different in Niger [41] and Nigeria [45], both of which are within the African meningitis belt, where some outbreaks with the rare serotype *N meningitidis* serogroup C were recorded. The serotype replacement can be attributed to the introduction of the meningococcal serogroup A conjugate vaccine (MenAfriVac) in most African countries [46].

 Meningitis requires accurate, prompt diagnosis and antibiotic therapy to ensure favorable outcomes. The reduced susceptibility of *S pneumoniae* to penicillin, with >40% strains with MIC bordering on the nonsusceptible range, suggests the need for prevention of this very serious condition by immunization and ongoing surveillance to monitor changes [8]. As seen in our study, the reduced susceptibility to penicillin and high susceptibility to ceftriaxone were similar to the findings in Mozambique [7] and Northern and Eastern African countries [47], which recorded medium to high resistance to penicillin and high susceptibility to ceftriaxone. These observations may be attributed to the longstanding use of penicillin as a first-line antibiotic for empiric treatment in most countries.

Limitations of this study were that the immunization history for Hib and PCV10 was not available. Patient outcome was not documented. Antimicrobial susceptibility testing was restricted to penicillin and ceftriaxone, and therefore changes in resistance to macrolides and other antibiotics, if any, are unknown. Antimicrobial susceptibility testing was performed on only 47 Spn isolates, 46 of which were from the prevaccine period; therefore, the susceptibility patterns of the postvaccine period was unknown. This study was limited to the children, whose parents gave consent, admitted to the UTH Children’s Hospital in Lusaka, which is a referral hospital and more densely populated, and therefore it may not be representative of the whole country.

## Conclusions

Our study has provided valuable information on the burden of vaccine-preventable IBDs. Although all 3 pathogens—*S pneumoniae*, *H influenzae*, and *N meningitidis*—were found to be causative agents of meningitis in children aged <5 years, *S pneumoniae* was still the leading cause, even in the face of high PCV10 coverage. A decrease in confirmed meningitis cases and PCV10 serotypes in the postvaccination period was consistent with a positive impact of PCV10 vaccination. However, the increase in *S pneumoniae* non-PCV10 serotypes warrants continued PBM surveillance to provide informed guidance on treatment and vaccination policies.

## Notes

***Acknowledgments.*** We acknowledge the Ministry of Health, The University Teaching Hospitals, Children’s’ Hospital, and the Bacteriology Unit under the Pathology and Microbiology department for providing a conducive working environment to enable this surveillance to take place. We also acknowledge the World Health Organization Regional Office for Africa and the National Institute for Communicable Diseases for their generous support.

***Disclaimer.*** The findings and conclusions in this report are those of the authors and do not represent the official position of the World Health Organization.

***Supplement sponsorship.*** This supplement is sponsored by the World Health Organization and the U. S. Centers for Disease Control and Prevention.

***Financial support.*** This work was funded by the Ministry of Health and the World Health Organization Regional Office for Africa. The Supplement was funded by the World Health Organization Regional Office for Africa.

***Potential conflict of interest.*** All authors: No reported conflicts of interest. All authors have submitted the ICMJE Form for Disclosure of Potential Conflicts of Interest.

## References

[1] UNICEF: WHO: World Bank: UN DESA. Levels & Trends in Child Mortality 2019. UN IGME Rep. 2019; 52.

[2] Jheeta M, Newell J. Childhood vaccination in Africa and Asia: the effects of parents’ knowledge and attitudes. Bull World Health Organ 2008; 86:419. 18568264 10.2471/BLT.07.047159 PMC2647458

[3] UNICEF. GIVS: global immunization vision and strategy, 2006–2015. Available at: www.miseenoeuvre.com. Accessed 21 December 2020.

[4] Oordt-Speets AM, Bolijn R, van Hoorn RC, Bhavsar A, Kyaw MH. Global etiology of bacterial meningitis: a systematic review and meta-analysis. PLoS One 2018; 13:e0198772. 29889859 10.1371/journal.pone.0198772 PMC5995389

[5] Wahl B, Sharan A, Deloria Knoll M, et al. National, regional, and state-level burden of *Streptococcus pneumoniae* and *Haemophilus influenzae* type b disease in children in India: modelled estimates for 2000–15. Lancet Glob Heal 2019; 7:e735–47. 10.1016/S2214-109X(19)30081-6 PMC6527518 31097277

[6] Lukwesa C, Mwansa J, Nakazwe R. Aetiological agents of meningitis in Zambia: is there a need for a pneumococcal vaccine? Int J Infect Dis 2012; 16:e229–30.

[7] Nhantumbo AA, Gudo ES, Caierão J, et al. Serotype distribution and antimicrobial resistance of *Streptococcus pneumoniae* in children with acute bacterial meningitis in Mozambique: implications for a national immunization strategy. BMC Microbiol 2016; 16:134. 27357587 10.1186/s12866-016-0747-y PMC4928344

[8] Mpabalwani EM, Lukwesa-Musyani C, Imamba A, et al. Declines in pneumonia and meningitis hospitalizations in children under 5 years of age after introduction of 10-valent pneumococcal conjugate vaccine in Zambia, 2010–2016. Clin Infect Dis 2019; 69:S58–65. 31505628 10.1093/cid/ciz456 PMC6761309

[9] Mwenda JM, Soda E, Weldegebriel G, et al. Clinical infectious diseases. Meningitis Surveill Africa 2019; 2019:49–57.

[10] Hausdorff WP, Feikin DR, Klugman KP. Epidemiological differences among pneumococcal serotypes. Lancet Infect Dis 2005; 5:83–93. 15680778 10.1016/S1473-3099(05)01280-6

[11] Wahl B, O’Brien KL, Greenbaum A, et al. Articles burden of *Streptococcus pneumoniae* and *Haemophilus**influenzae* type b disease in children in the era of conjugate vaccines: global, regional, and national estimates for 2000–15. The Lancet Global Health 2019; 7:e735–47.31097277

[12] Edmond K, Clark A, Korczak VS, Sanderson C, Griffiths UK, Rudan I. Global and regional risk of disabling sequelae from bacterial meningitis: a systematic review and meta-analysis. Lancet Infect Dis 2010; 10:317–28. 20417414 10.1016/S1473-3099(10)70048-7

[13] Slack M, Esposito S, Haas H, et al. *Haemophilus influenzae* type b disease in the era of conjugate vaccines: critical factors for successful eradication. Expert Rev. Vaccines [Internet] 2020; 19:903–17. Available at: 10.1080/14760584.2020.1825948 32962476

[14] Watt JP, Wolfson LJ, O’Brien KL, et al.; Hib and Pneumococcal Global Burden of Disease Study Team. Burden of disease caused by *Haemophilus influenzae* type b in children younger than 5 years: global estimates. Lancet 2009; 374:903–11. 19748399 10.1016/S0140-6736(09)61203-4

[15] Gabutti G, Stefanati A, Kuhdari P. Epidemiology of *Neisseria meningitidis* infections: case distribution by age and relevance of carriage. J Prev Med Hyg 2015; 56:E116–20. 26788731 PMC4755119

[16] Vuocolo S, Balmer P, Gruber WC, et al. Vaccination strategies for the prevention of meningococcal disease. Hum Vaccines Immunother 2018; 14:1203–15. 10.1080/21645515.2018.1451287 PMC5989901 29543535

[17] Balmer P, Burman C, Serra L, York LJ. Impact of meningococcal vaccination on carriage and disease transmission: a review of the literature. Hum Vaccines Immunother 2018; 14:1118–30. 10.1080/21645515.2018.1454570 PMC5989891 29565712

[18] WHO. Recommendations for Interrupted or Delayed Routine Immunization - Summary of WHO Position Papers [Internet]. 2015. Available at: http://www.who.int/immunization/policy/Immunization. Accessed 16 March 2021.

[19] Davis S, Feikin D, Johnson HL. The effect of Haemophilus influenzae type B and pneumococcal conjugate vaccines on childhood meningitis mortality: a systematic review. BMC Public Health [Internet] 2013; 13:S21. Available at: http://www.biomedcentral.com/1471-2458/13/S3/S21. Accessed 7 February 2020. 10.1186/1471-2458-13-S3-S21 PMC3847464 24564188

[20] World Health Organization. Vaccine Preventable Diseases Surveillance Standards [Internet]. WHO, 2020 [cited 2021 Jun 8]; Available at: https://www.who.int/publications/m/item/vaccine-preventable-diseases-surveillance-standards-pneumococcus. Accessed 12 January 2021.

[21] World Health Organization. Vaccine Preventable Diseases Surveillance Standards [Internet]. WHO, 2020 [cited 2021 Jun 7]. Available at: http://www.who.int/immunization/monitoring_surveillance/burden/vpd/standards/en/. Accessed 7 June 2021.

[22] World Health Organization. Bacterial meningitis (including Haemophilus influenzae type b (Hib), Neisseria meningitidis, and Streptococcus pneumoniae). Available at: http://www.who.int/immunization/monitoring_surveillance/burden/vpd/surveillance_type/sentinel/meningitis_surveillance/en/. Accessed 15 March 2021.

[23] Wang X, Theodore MJ, Mair R, et al. Clinical validation of multiplex real-time PCR assays for detection of bacterial meningitis pathogens. J Clin Microbiol 2012; 50:702–8. 22170919 10.1128/JCM.06087-11 PMC3295090

[24] Magomani V, Wolter N, Tempia S, du Plessis M, de Gouveia L, von Gottberg A. Challenges of using molecular serotyping for surveillance of pneumococcal disease. J Clin Microbiol 2014; 52:3271–6. 24958802 10.1128/JCM.01061-14 PMC4313149

[25] Pimenta FC, Roundtree A, Soysal A, et al. Sequential triplex real-time PCR assay for detecting 21 pneumococcal capsular serotypes that account for a high global disease burden. J Clin Microbiol 2013; 51:647–52. 23224094 10.1128/JCM.02927-12 PMC3553924

[26] Wang X, Theodore MJ, Mair R, et al. Clinical validation of multiplex real-time PCR assays for detection of bacterial meningitis pathogens. J Clin Microbiol 2012; 50:702–8. 22170919 10.1128/JCM.06087-11 PMC3295090

[27] Maaroufi Y, De Bruyne JM, Heymans C, Crokaert F. Real-time PCR for determining capsular serotypes of *Haemophilus influenzae*. J Clin Microbiol 2007; 45:2305–8. 17507524 10.1128/JCM.00102-07 PMC1932976

[28] CLSI. M07: dilution AST for aerobically grown bacteria. Available at: https://clsi.org/standards/products/microbiology/documents/m07/. Accessed 14 April 2020.

[29] Munguambe AM, De Almeida AECC, Nhantumbo AA, et al. Characterization of strains of *Neisseria meningitidis* causing meningococcal meningitis in Mozambique, 2014: implications for vaccination against meningococcal meningitis. PLoS One 2018; doi:10.1371/journal.pone.0197390. PMC6082507 30089105

[30] von Mollendorf C, Tempia S, von Gottberg A, et al. Estimated severe pneumococcal disease cases and deaths before and after pneumococcal conjugate vaccine introduction in children younger than 5 years of age in South Africa. PLoS One 2017; 12:e0179905. 28671978 10.1371/journal.pone.0179905 PMC5495214

[31] Oordt-Speets AM, Bolijn R, Van Hoorn RC, Bhavsar A, Kyaw MH. Global etiology of bacterial meningitis: a systematic review and meta-analysis. PLoS One 2018; doi:10.1371/journal.pone.0198772 PMC5995389 29889859

[32] Cornick JE, Everett DB, Broughton C, et al. Invasive *Streptococcus pneumoniae* in children, Malawi, 2004–2006. Emerg Infect Dis 2011; 17:1107–9. 21749782 10.3201/eid1706.101404 PMC3358202

[33] Tan TQ. Pediatric invasive pneumococcal disease in the United States in the era of pneumococcal conjugate vaccines. Clin Microbiol Rev 2012; 25:409–19. 22763632 10.1128/CMR.00018-12 PMC3416489

[34] Tagbo BN, Bancroft RE, Fajolu I, Abdulkadir MB, Bashir MF, Okunola OP, et al. Pediatric Bacterial Meningitis Surveillance in Nigeria from 2010 to 2016, Prior to and during the Phased Introduction of the 10-Valent Pneumococcal Conjugate Vaccine. Clin. Infect. Dis. 2019; 69:S81–8. 31505626 10.1093/cid/ciz474 PMC6736152

[35] Nhantumbo AA, Weldegebriel G, Katsande R, et al. Surveillance of impact of PCV-10 vaccine on pneumococcal meningitis in Mozambique, 2013 - 2015. PLoS One 2017; doi: 10.1371/journal.pone.0177746. PMC5467806 28604773

[36] Levy C, Ouldali N, Caeymaex L, Angoulvant F, Varon E, Cohen R. Diversity of serotype replacement after pneumococcal conjugate vaccine implementation in Europe. J Pediatr 2019; 213:252–53.e3. 31561776 10.1016/j.jpeds.2019.07.057

[37] Madhi SA, Nunes MC. The potential impact of pneumococcal conjugate vaccine in Africa: considerations and early lessons learned from the South African experience. Hum Vaccin Immunother 2016; 12:314–25. 26317537 10.1080/21645515.2015.1084450 PMC5049711

[38] Løchen A, Croucher NJ, Anderson RM. Divergent serotype replacement trends and increasing diversity in pneumococcal disease in high income settings reduce the benefit of expanding vaccine valency. Sci Rep 2020; 10:18977. 33149149 10.1038/s41598-020-75691-5 PMC7643077

[39] World Health Organization (WHO). Global strategy on comprehensive vaccine-preventable disease surveillance [Internet]. 2020 [cited 2021 Jun 9]. Available at: https://www.technet-21.org/en/library/main/6641-global-strategy-on-comprehensive-vaccine-preventable-disease-surveillance. Accessed 9 June 2021.

[40] Sanneh B, Okoi C, Grey-Johnson M, et al. Declining trends of pneumococcal meningitis in Gambian children after the introduction of pneumococcal conjugate vaccines. Clin Infect Dis 2019; 69:126–32. 10.1093/cid/ciz505 PMC6761313 31505634

[41] Sidikou F, Zaneidou M, Alkassoum I, et al.; MenAfriNet consortium. Emergence of epidemic *Neisseria meningitidis* serogroup C in Niger, 2015: an analysis of national surveillance data. Lancet Infect Dis 2016; 16:1288–94. 27567107 10.1016/S1473-3099(16)30253-5 PMC5737706

[42] von Gottberg A, du Plessis M, Cohen C, et al.; Group for Enteric, Respiratory and Meningeal Disease Surveillance in South Africa. Emergence of endemic serogroup W135 meningococcal disease associated with a high mortality rate in South Africa. Clin Infect Dis 2008; 46:377–86. 18181736 10.1086/525260

[43] Rasoanandrasana S, Raberahona M, Milenkov M, et al. Resurgence of *Neisseria meningitidis* serogroup W ST-11 (cc11) in Madagascar, 2015–2016. Int J Infect Dis 2017; 55:1–3. 27940178 10.1016/j.ijid.2016.12.001

[44] Ibarz-Pavón AB, Morais L, Sigaúque B, et al. Epidemiology, molecular characterization and antibiotic resistance of *Neisseria meningitidis* from patients ≤15 years in Manhiça, rural Mozambique. PLoS One 2011; 6:e19717. 21695194 10.1371/journal.pone.0019717 PMC3112148

[45] Nnadi C, Oladejo J, Yennan S, et al. Large outbreak of *Neisseria meningitidis* Serogroup C — Nigeria, December 2016–June 2017. Morb Mortal Wkly Rep 2017; 66:1352–6. 10.15585/mmwr.mm6649a3 PMC5730219 29240724

[46] Bwaka A, Bita A, Lingani C, et al. Status of the rollout of the meningococcal serogroup A conjugate vaccine in African meningitis belt countries in 2018. J Infect Dis 2019; 220:140–7. 10.1093/infdis/jiz336 PMC6822965 31671448

[47] Iroh Tam PY, Thielen BK, Obaro SK, et al. Childhood pneumococcal disease in Africa - A systematic review and meta-analysis of incidence, serotype distribution, and antimicrobial susceptibility. Vaccine 2017; 35:1817–27. 28284682 10.1016/j.vaccine.2017.02.045 PMC5404696

